# Evidence for de novo acquisition of microalgal symbionts by bleached adult corals

**DOI:** 10.1038/s41396-022-01203-0

**Published:** 2022-02-07

**Authors:** Hugo J. Scharfenstein, Wing Yan Chan, Patrick Buerger, Craig Humphrey, Madeleine J. H. van Oppen

**Affiliations:** 1 School of BioSciences, The University of Melbourne, Parkville, VIC Australia; 2 Australian Institute of Marine Science, Townsville, QLD Australia; 3 Applied BioSciences, Macquarie University, Sydney, NSW Australia

**Keywords:** Symbiosis, DNA sequencing

## Abstract

Early life stages of most coral species acquire microalgal endosymbionts (Symbiodiniaceae) from the environment, but whether exogenous symbiont uptake is possible in the adult life stage is unclear. Deep sequencing of the Symbiodiniaceae ITS2 genetic marker has revealed novel symbionts in adult corals following bleaching; however these strains may have already been present at densities below detection limits. To test whether acquisition of symbionts from the environment occurs, we subjected adult fragments of corals (six species in four families) to a chemical bleaching treatment (menthol and DCMU). The treatment reduced the native microalgal symbiont abundance to below 2% of their starting densities. The bleached corals were then inoculated with a cultured *Cladocopium* C1^acro^ strain. Genotyping of the Symbiodiniaceae communities before bleaching and after reinoculation showed that fragments of all six coral species acquired the *Cladocopium* C1^acro^ strain used for inoculation. Our results provide strong evidence for the uptake of Symbiodiniaceae from the environment by adult corals. We also demonstrate the feasibility of chemical bleaching followed by reinoculation to manipulate the Symbiodiniaceae communities of adult corals, providing an innovative approach to establish new symbioses between adult corals and heat-evolved microalgal symbionts, which could prove highly relevant to coral reef restoration efforts.

Scleractinian corals prosper in oligotrophic waters by forming mutualistic relationships with microalgae (Symbiodiniaceae) that translocate photosynthate to their host [1]. Breakdown of the coral-Symbiodiniaceae symbiosis, i.e. coral bleaching, occurs in response to environmental stress and may result in widespread coral mortality [2]. Thermal stress is the primary cause of large-scale coral bleaching events, which have become more frequent and severe over recent years as climate change-driven marine heatwaves gain prevalence [3].

Bleaching tolerance of corals to elevated temperatures varies within and among species. This is partly determined by the physiological performances of their microalgal symbionts under thermal stress [4, 5]. For instance, thermotolerant Symbiodiniaceae species in the genus *Durusdinium* have been found to increase the bleaching threshold of the coral holobiont by 1–2 °C [6].

The Symbiodiniaceae comprise at least 15 genera and genus-level lineages which include many species [7]. Several microalgal symbionts may coexist within a coral host, with the Symbiodiniaceae communities of adult corals often being dominated by a single species [8]. Other members of the symbiont community are found in low abundances, forming a Symbiodiniaceae rare biosphere increasingly linked to coral bleaching resilience [9]. Changes in the relative abundance of Symbiodiniaceae species (shuffling) [10] and the acquisition of new symbionts from the environment (switching) [11] in adulthood are potential mechanisms for corals to adapt to increases in sea surface temperatures [4–6, 8–11]. Evidence of symbiont shuffling is widespread [4, 5, 10], yet reports of switching in adult corals remain limited to metabarcoding studies [12] and the Symbiodiniaceae rare biosphere [11], where the acquired symbionts may have been present below the detection limit before bleaching. Whilst environmental uptake of exogenous Symbiodiniaceae has been demonstrated in adult sea anemones [13] and octocorals [14], experimental evidence of switching in adult corals remains unconvincing with only a transient symbiosis being reported in adult colonies of *Porites divarcata* following bleaching [15].

Here we used chemical bleaching to remove >98% of the native symbiont cells from adult fragments of six coral species spanning four families (i.e. *Diploastrea heliopora* (Diploastraeidae)*, Dipsastraea pallida* (Merulinidae)*, Echinopora lamellosa* (Merulinidae)*, Platygyra daedalea* (Merulinidae)*, Porites lobata* (Poritidae) and *Stylophora pistillata* (Pocilloporidae)), which were then successfully reinfected with a cultured Symbiodiniaceae strain. Colonies were fragmented and chemically bleached (*n* = 16 per species) through exposure to menthol and 3-(3,4-dichlorophenyl)-1,1-dimethylurea (DCMU; Supplementary methods section 1.3). Once bleached, the coral fragments underwent four different reinoculation treatments (*n* = 3 per species and treatment): (1) a negative control treatment (Ctl-) where corals were not reinoculated with any Symbiodiniaceae; (2) a positive control treatment (Ctl+) where corals were reinoculated with freshly isolated homologous Symbiodiniaceae (obtained through tissue blasting of conspecific coral fragments); (3) a reinoculation treatment (Ri) where corals were reinoculated with a cultured *Cladocopium* C1^acro^ strain (SCF055-01.10) at 10^4^ cells/ml; (4) a reinoculation with sand treatment (RiS) where corals were reinoculated as in treatment Ri in the presence of sterilised sand (Supplementary methods 1.4).

Coral pigmentation, a phenotypic trait partly dependent on Symbiodiniaceae abundance, was scored to assess the progression of bleaching and symbiosis establishment in the corals following the methodology established by Quigley et al. [16]; see Supplementary methods 1.5. Symbiodiniaceae cell density *in hospite* was quantified before and after chemical bleaching, and nine weeks after the first reinoculation to assess the extent of bleaching and recovery (Supplementary methods 1.6).

A decrease in pigmentation and a 98.3–99.8% reduction in Symbiodiniaceae density between the start and end of chemical bleaching was recorded in all six coral species (Figs. 1A, B; S1). Following reinoculation with the *Cladocopium* C1^acro^ strain, re-pigmentation was observed across all six coral species (Figs. 1B; S2). Pigmentation scores and symbiont densities were higher in treatments Ri and RiS than in treatment Ctl-, except for *P. daedalea* which repigmented equally across all treatments (Figs. S2, S3).

**Fig. 1:**
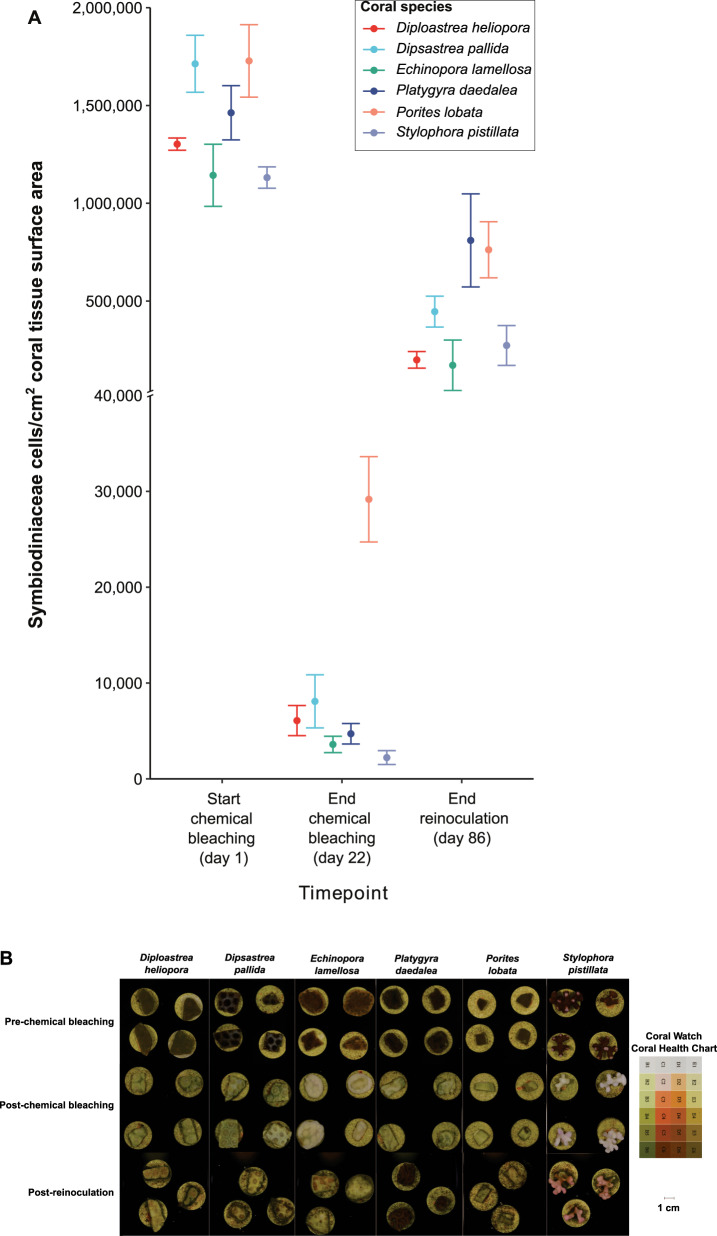
Coral bleaching and repigmentation responses to chemical bleaching and reinoculation. **A** Symbiodiniaceae cell densities *in hospite* before and after chemical bleaching (*n* = 4 per species) and after reinoculation (*n* = 6 per species, except for *E. lamellosa*: *n* = 2; *P.daedalea*: *n* = 5; *S. pistillata*: *n* = 5). Error bars represent 1 standard error. **B** Images of corals before and after chemical bleaching and nine weeks after reinoculation with a cultured *Cladocopium* C1^acro^ strain in the presence of sterilised sand (treatment RiS shown here). Both *D. pallida* and D*. heliopora* displayed thinner tissue after reinoculation resulting in their septa standing out. No tissue necrosis was recorded in these coral species, contrarily to *E. lamellosa* and *P. lobata* fragments. The Coral Watch Coral Health Chart depicts differences in pigmentation when a coral undergoes bleaching.

Sequencing of the Internal Transcribed Spacer 2 (ITS2) region of the Symbiodiniaceae nrDNA was undertaken to characterise the symbiont communities before bleaching and nine weeks after the first reinoculation with *Cladocopium* C1^acro^ (Table S1; Supplementary methods 1.6). The native Symbiodiniaceae communities of the six coral species were genetically distinct from the *Cladocopium* C1^acro^ phylotype used for reinoculation (Fig. 2). In all six coral species, the native Symbiodiniaceae were replaced with the inoculum *Cladocopium* C1^acro^ in treatments Ri and RiS, while this *Cladocopium* C1^acro^strain was not detected in the native symbiont communities of the corals (Figs. 2, S3–S4; Table S2), demonstrating that severely bleached adult corals are able to acquire new symbionts from the environment.

**Fig. 2:**
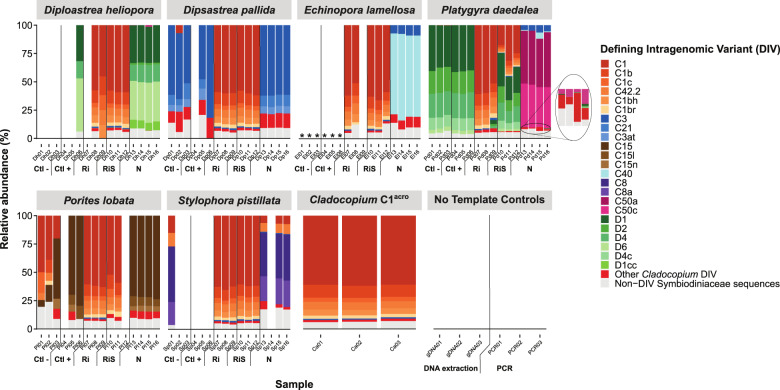
Relative abundance of Symbiodiniaceae community (ITS2) profiles. Symbiodiniaceae community profiles in the six coral species before bleaching and 9 weeks after inoculation and the cultured *Cladocopium* C1^acro^ strain used for reinoculation. Defining intragenomic variants (DIVs) are recurring assemblages of ITS2 sequences found at set abundances across samples. Each bar represents a sample from a coral fragment (*n* = 3 per reinoculation treatment, *n* = 4 for treatment N), with each individual bar representing the proportion of a DIV sequence relative to the total abundance of sequences. Empty bars correspond to samples for which no reads were obtained or which were removed for possessing only one DIV (considered as insufficient sequence depth). No-template controls from the DNA extraction and PCR amplification (*n* = 3 for each) were also sequenced and are shown. Ctl− = negative control treatment where corals were chemically bleached but not reinoculated with any Symbiodiniaceae. Ctl+ = positive control treatment where corals were chemically bleached and then reinoculated with freshly isolated homologous Symbiodiniaceae. RI = reinoculation treatment where corals were reinoculated with a cultured *Cladocopium* C1^acro^ strain. RiS = reinoculation with sand treatment where corals were reinoculated as in treatment Ri in the presence of sterilised sand. N = native Symbiodiniaceae community before bleaching. * = dead corals.

The environmental acquisition of the *Cladocopium* C1^acro^ strain by *P. lobata* and *S. pistillata* contributes to the growing body of evidence that vertically transmitting corals also possess the ability to acquire symbionts horizontally [17]. The presence of sand during reinoculation had a measurable effect on the composition of the Symbiodiniaceae community in *P. daedalea* only, which led to a mixed community with a *Durusdinium* phylotype that was initially detected in low abundance (<1%).

Chemical bleaching is increasingly used in studies investigating cnidarian-symbiont interactions to obtain aposymbiotic hosts for reinfection with cultured Symbiodiniaceae [13, 18]. Adult *Stylophora pistillata* and *Isopora palifera* corals have been found to lose 99% of their algal symbiont densities following menthol exposure [19]. Comparable levels of Symbiodiniaceae density reduction (98.3–99.8%) were observed for the six coral species used here. Unlike *Exaiptasia diaphana* [18], none of the coral species in this study were rendered completely aposymbiotic from exposure to menthol and DCMU. Severe reduction of the native Symbiodiniaceae communities, rather than complete elimination, is thus sufficient for novel symbioses to establish, though the long-term stability (>9 weeks after reinoculation) of these symbioses needs to be investigated.

Our findings provide compelling evidence that adult fragments of six coral species, spanning four families in the complex (*P. lobata*) and robust (*D. heliopora, S. pistillata, D. pallida, E. lamellosa, P. daedalea*) clades, can acquire heterologous *Cladocopium* C1^acro^ symbionts from the environment. The chemical bleaching and reinoculation methodology may prove highly relevant for the study of coral-Symbiodiniaceae interactions and for the development of adult coral stock with enhanced thermal tolerance for reef restoration by inoculating them with heat-evolved Symbiodiniaceae [20].

## Supplementary information

Supplementary Information

Dataset 1

## Data Availability

Raw sequences of the ITS2 Symbiodiniaceae datasets are available in GenBank (BioSample accession: SAMN22047733 – SAMN22047828; BioProject ID: PRJNA768535).
