# Supplementary material for: Evidence for de novo acquisition of microalgal symbionts by bleached adult corals
Source: ISME J. 2022 Feb 7;16(6):1676–9. doi: 10.1038/s41396-022-01203-0 (PMC9122906; doi:10.1038/s41396-022-01203-0)
Supplement: Supplementary file 1 — Supplementary Information [file 41396_2022_1203_MOESM1_ESM.docx]

**Supplementary Information**

1. **Materials and Methods**
   1. **Coral sampling and fragmentation**

A single colony from each adult coral species was collected on 29.06.2020 between depths of 5-10 m from Davies Reef in the central region of the Great Barrier Reef (permit collection n°: G12/35236.1) and transported to aquarium facilities at the National Sea Simulator (Australian Institute of Marine Science). The colonies were fragmented into nubbins (n=20 per coral species) using a band saw. A minimum of 2-3 polyps per nubbin were kept for the large-polyp coral species (*Diploastrea heliopora, Dipsastrea pallida, Echinopora lamellosa*) and of 1.5 x 1.5 cm for small-polyp species (*Porites lobata, Stylophora pistillata*) and the brain coral (*Platygyra daedalea*). The nubbins were then superglued to aragonite plugs (Frag Plugs Aragonite Large; OW100LCFP, Aquasonic, Wauchope, Australia). The coral fragments were subsequently left to recover in their experimental tanks for four weeks in filtered seawater (1 µm; FSW) with daily feeding of *Artemia nauplii* at 0.5 nauplii/ml. Water temperature and lighting conditions were identical to experimental conditions (see below).

- 1. **Experimental conditions**

For each coral species, 20 nubbins were evenly distributed between four tanks. Hydra 64HD lights (Aqua Illumination, Bethlehem, PA, USA) were used for illumination. The light intensity for all tanks was between 82-180 µmol/m^2^/s. The coral nubbins were cleaned 1-2 times a week to remove filamentous algae, after which the corals were moved around the tanks. Water temperature was maintained between 26.7-27°C for the duration of the experiment. Air stones were used for water movement and aeration to avoid dissolved oxygen depletion during chemical bleaching and reinoculation. The photoperiod of the corals was 11h (6:50-17:50).

- 1. **Chemical bleaching procedure**

The chemical bleaching procedure used was based on the methodology established by Matthews *et al*. 2016 and Wang *et al*. 2012 [1,2]. Menthol (M2772, Sigma-Aldrich, St. Louis, MO, USA) and 3-(3,4-dichlorophenyl)-1,1-dimethylurea (DCMU; D2425, Sigma-Aldrich) were added to 1 l of FSW at a final concentration of 0.39 mM and 0.13 µM, respectively. The coral fragments were kept in the menthol and DCMU spiked seawater for 8 h (8:30-16:30), corresponding to most of the photoperiod. The seawater was then fully replaced and DCMU was added at 0.13 µM for 16h (16:30-8:30, overnight). This procedure was repeated over four consecutive days, after which the corals were returned to flowing seawater for a 3-day recovery period. This weekly cycle was repeated three times consecutively. No feeding took place during the 3-week chemical bleaching period.

- 1. **Reinoculation procedure**

Coral fragments from treatments Ctl+, Ri and RiS were reinoculated and fed simultaneously with *Artemia nauplii* at a density of 0.5 nauplii/ml to elicit a feeding response. Corals from treatment Ctl- were fed the same amount and at the same time, though no reinoculation was carried out. For treatments Ri and RiS, strain SCF055-01.10 from the Symbiont Culture Facility (AIMS) was used, which is closely related to *Cladocopium goreaui* and referred here as *Cladocopium* C1^acro^ strain following Beltran *et al.* [3]. The sand used in treatment RiS, which originated from Davies Reef, was sieved through a 1 mm sieve and sterilised through autoclaving. Enough sand to cover the bottom of a tank (corresponding to a volume of 100 ml per tank) was added to the tanks before inoculation. The addition of sand in treatment RiS was intended to aid with the uptake of the *Cladocopium* C1^acro^ strain since Symbiodiniaceae are known to proliferate in sediment [4]. Corals were incubated in 1 l of FSW with their inoculum and *Artemia* for 16h (overnight) before being returned to flowing FSW. This procedure was repeated for four days followed by 3-day recovery period in flowing FSW, during which feeding was maintained. This weekly cycle was repeated twice consecutively. The corals were maintained under the experimental conditions described in section 1.2. for the remainder of the experiment.

- 1. **Visual scoring of coral pigmentation**

Visual scoring of coral pigmentation was assessed following the methodology that was established by Quigley *et al.* [5]. The non-invasive nature of pigmentation scoring enabled bi-weekly measurements during chemical bleaching. Briefly, this method consists of calculating the average RGB (red, green, blue) brightness values of the coral nubbins using ImageJ. RGB brightness was calibrated using the Coral Watch Coral Health Chart categories D1 through D6 (or C1 - C6 or E1 -E6 or B1 - B6 depending on the closest matching colour for the pigmentation of the coral species). These categories represent the varying degrees of pigmentation of a coral when it undergoes bleaching [6]. Linear equations were calculated for each photo by regressing the corresponding category RGB measurements with their colour score (1 - 6). A score of 1 corresponding to a bleached coral and a score of 6 corresponding to a healthy coral (values greater than 6 were assigned a score of 6). The linear equation was used to obtain a pigmentation score from the average RGB brightness of the entire live tissue from a coral nubbin.

- 1. **Symbiodiniaceae cell density quantification *in hospite***

Coral fragments were sampled before and after chemical bleaching and nine weeks after the first reinoculation for Symbiodiniaceae cell density quantification. This procedure was based on the methodology developed by McCowan *et al*. 2011 [7]. Entire coral fragments were snap frozen in liquid nitrogen and stored at -80°C. Immediately after removal from storage the corals were fixed for seven days in 10 % (v/v) formalin using 0.22 µm FSW, following which the corals were decalcified in 5 % (v/v) hydrochloric acid. The hydrochloric acid was refreshed every 4-5 days until the coral tissue separated from the skeleton. The coral tissue was then homogenised through bead beating at 4 m/s for 20 s and sonicated for 20 s. The homogenate subsequently underwent three washes, each wash consisting of centrifugation (3 min x 3 000 g) and resuspension in 0.22 µm FSW. A Bright-Line™ Haemacytometer (Sigma-Aldrich) was used to count Symbiodiniaceae cells (8 replicate counts per sample). The counts were normalised to the coral tissue surface area, which was measured through photogrammetry following the process described by Figueira *et al*. 2015 [8].

- 1. **ITS2 metabarcoding**

Coral fragments were sampled before chemical bleaching and nine weeks after the first reinoculation for Symbiodiniaceae metabarcoding. If coral fragments displayed insufficient live coral tissue for sampling for Symbiodiniaceae cell density quantification and ITS2 metabarcoding, then ITS2 metabarcoding was prioritised. Coral fragments were snap frozen in liquid nitrogen and stored at -80°C. DNA extraction was performed following the methodology detailed by Wilson *et al*. 2002 [9]. PCR amplification of the ITS2 region was carried out in triplicate using the SYM_VAR_5.8S2 (forward: 5’-GTGACCTATGAACTCAGGAGTCGAATTGCAGAACTCCGTGAACC-3’) and SYM_VAR_REV (reverse: 5’-CTGAGACTTGCACATCGCAGCCGGGTTCWCTTGTYTGACTTCATGC-3’) primers developed by Hume *et al*. 2018 [10] with Illumina adapters underlined. PCR reactions were set up with: 30 µl Taq PCR Master Mix (201445, Qiagen, Hilden, Germany), 3 µl DNA template, 1.5 µl of each primer (10 µM working solution) and 24 µl Milli-Q ultrapure water. The thermocycling consisted of an initial denaturation step at: 95.0°C for 15 min, at 95°C for 15 s, 56°C and 72°C for 30 s each (18 cycles), and 72°C for 7 min. The triplicate PCR reactions were pooled for library preparation, which was carried out in accordance to Dungan *et al*. 2021 [11]. The samples were sequenced on an Illumina MiSeq v3 platform (2x300 bp) at the Walter and Eliza Hall Institute, Melbourne, Australia.

- 1. **SymPortal analysis**

The demultiplexed raw sequences were submitted to SymPortal for ITS2 profiling [12]. The presence and abundance of recurring assemblages of ITS2 sequences across samples, referred to as defining intragenomic variants (DIVs), are considered by SymPortal to identify ITS2 profiles, which are representative of putative Symbiodiniaceae taxa. Sequences that are not identified as DIVs (non-DIV sequences) are not assigned to an ITS2 profile. Replicates that possessed a single DIV were considered to have an insufficient sequence depth and were removed. Samples possessing a low sequence depth (<5k) were kept if they possessed multiple DIVs. No rarefaction of the sequences was carried out to avoid omission of rarer sequences [13], particularly since coral nubbins from treatments Ctl- and Ctl+ possessed a low sequencing read depth and because the main objective of the analysis was looking at the presence or absence of Symbiodiniaceae strains.

**References**

1. Matthews JL, Sproles AE, Oakley CA, Grossman AR, Weis VM, Davy SK. Menthol-induced bleaching rapidly and effectively provides experimental aposymbiotic sea anemones (*Aiptasia* sp.) for symbiosis investigations. *J. Exp. Biol*. 2016; 219: 306–310.
2. Wang JT, Chen YY, Tew KS, Meng PJ, Chen CA. Physiological and Biochemical Performances of Menthol-Induced Aposymbiotic Corals. *PLoS ONE*. 2012; 7: e46406.
3. Beltran VH, Puill-Stephan E, Howells E, Flores-Moya A, Doblin M, Nuñez-Lara E. *et al.* Physiological diversity among sympatric, conspecific endosymbionts of coral (*Cladocopium* C1^acro^) from the Great Barrier Reef. *Coral Reefs*. 2021; 40, 985-997.
4. Nitschke MR, Davy SK, Ward S. Horizontal transmission of *Symbiodinium* cells between adult and juvenile corals is aided by benthic sediment. *Coral Reefs*. 2016; 35: 335-344.
5. Quigley KM, Willis BL, Kenkel CD. Transgenerational inheritance of shuffled symbiont communities in the coral *Montipora digitata*. *Sci. Rep*. 2019; 9: 13328.
6. Siebeck UE, Marshall NJ, Klüter A, Hoegh-Goldberg O. Monitoring coral bleaching using a colour reference card. *Coral Reefs.* 2006; 25: 453-460.
7. McCowan DM, Pratchett MS, Paley AS, Seeley M, Baird AH. A comparison of two methods of obtaining densities of zooxanthellae in *Acropora millepora. Galaxea, JCRS*. 2011; 13: 29-34.
8. Figueira W, Renata F, Weatherby E, Porter A, Hawes S, Byrne M. Accuracy and Precision of Habitat Structural Complexity Metrics Derived from Underwater Photogrammetry. *Remote Sens*. 2015; 7: 16883-16900.
9. Wilson K, Li Y, Whan V, Lehnert S, Byrne K, Moore S, Pongsomboon S, Tassanakajon A, Rosenberg G, Ballment E, Fayazi Z, Swan J, Kenway M, Benzie J. Genetic mapping of the black tiger shrimp *Penaeus monodon* with amplified fragment length polymorphism. *Aquaculture*. 2002; 204: 297–309.
10. Hume BCC, Ziegler M, Poulain J, Pochon X, Romac S, Boissin E *et al*. An improved primer set and amplification protocol with increased specificity and sensitivity targeting the *Symbiodinium* ITS2 region. *PeerJ*. 2018; 6: e4816.
11. Dungan AM, van Oppen MJH, Blackall LL. Short-Term Exposure to Sterile Seawater Reduces Bacterial Community Diversity in the Sea Anemone, *Exaiptasia diaphana*. *Front. Mar. Sci.* 2021; 7: 599314.
12. Hume BCC, Smith ES, Ziegler M, Warrington HJM, Burt JA, LaJeunesse TC, Wiedenmann J, Voolstra R. SymPortal: A novel analytical framework and platform for coral algal symbiont next-generation sequencing ITS2 profiling. *Mol Ecol Resour*. 2019; 19: 1063–1080.
13. McMurdie PJ, Holmes S. Waste Not, Want Not: Why Rarefying Microbiome Data Is Inadmissible. *PLoS Comput. Biol*. 2014; 10: e1003531.
14. **Supplementary tables and figures**

**Table S1: Sequencing read depth and number of DIVs across samples processed by SymPortal**. ^a^ = Samples that possessed a single DIV which were removed from our analysis as these were considered to be insufficiently sequenced. ^b^ = Samples for which DIVs were identified by SymPortal but failed to meet the minimum sequencing depth threshold for constructing ITS2 type profiles. Ctl - = negative control treatment where corals were chemically bleached but not reinoculated with any Symbiodiniaceae. Ctl + = positive control treatment where corals were chemically bleached and then reinoculated with freshly isolated homologous Symbiodiniaceae. RI = reinoculation treatment where corals were reinoculated with a cultured *Cladocopium* C1^acro^ strain. RiS = reinoculation with sand treatment where corals were reinoculated as in treatment Ri in the presence of sterilised sand. N = native Symbiodiniaceae community before bleaching. * = dead corals.

**Table S2: Average sequencing read depth of the DIVs composing the ITS2 type profile of the *Cladocopium* C1^acro^ strain in the samples from the native symbiont communities of all six coral species.**

**Figure S1: Coral pigmentation scores.** Pigmentation scores for fragments from all six coral species during chemical bleaching (A) and nine weeks after the first reinoculation across all four reinoculation treatments (B). Replication per coral species for A: n = 4. Replication per coral species/treatment for B: n = 3 (except for *E. lamellosa*/Ri: n = 1; *P. lobata*/Ctl -: n = 1). Error bars represent 1 standard error. * = dead corals.

**Figure S2: Symbiodiniaceae cell densities *in hospite* nine weeks after reinoculation.** Symbiodiniaceae cell densities *in hospite* obtained from haemocytometer counts for all six coral species across the four reinoculation treatments. Replication per coral species/treatment: n = 3 (except for *D. heliopora*/Ctl+: n = 2; *E. lamellosa*/RiS: n = 2; *P.daedalea*/Ctl-: n = 1; *P.daedalea*/Ctl+: n = 2; *P.daedalea*/Ri: n = 2; *P. lobata*/Ctl+: n = 1; *S. pistillata*/Ctl-: n = 2; *S. pistillata*/RiS: n = 2). No cell density measurements were carried out for *E. lamellosa*/Ri, *P. lobata*/Ctl- and *P. lobata*/Ctl+ due to insufficient live coral tissue being available for Symbiodiniaceae cell density quantification. Error bars represent 1 standard error. * = dead corals.

**Figure S3: Relative abundances of Symbiodiniaceae (ITS2) profiles.** ITS2 profiles of the Symbiodiniaceae communities from all six coral species generated by SymPortal. Each stacked column bar represents the proportion of ITS2 type profiles identified by SymPortal in each sample. Relative abundances are inferior to 1 due to the presence of sequences that were not identified as part of any ITS2 profile (non-DIV sequences). Each bar represents a sample from a coral fragment (n=3 per reinoculation treatment, n=4 for treatment N). Empty bars correspond to samples which failed to meet SymPortal’s minimum sequence depth threshold for constructing ITS2 type profiles. No-template controls from the DNA extraction and PCR amplification (n=3 for each) were also sequenced and are shown. Ctl - = negative control treatment where corals were chemically bleached but not reinoculated with any Symbiodiniaceae. Ctl + = positive control treatment where corals were chemically bleached and then reinoculated with freshly isolated homologous Symbiodiniaceae. RI = reinoculation treatment where corals were reinoculated with a cultured *Cladocopium* C1^acro^ strain. RiS = reinoculation with sand treatment where corals were reinoculated as in treatment Ri in the presence of sterilised sand. N = native Symbiodiniaceae community before bleaching. * = dead corals.

**Figure S4**: **Enlarged representation of the** **relative abundance of Symbiodiniaceae community (ITS2) profiles.** Symbiodiniaceae community profiles in the six coral species before bleaching and nine weeks after inoculation and the cultured *Cladocopium* C1^acro^ strain used for reinoculation. Defining intragenomic variants (DIVs) are recurring assemblages of ITS2 sequences found at set abundances across samples. Each bar represents a sample from a coral fragment (n=3 per reinoculation treatment, n=4 for treatment N), with each individual bar representing the proportion of a DIV sequence relative to the total abundance of sequences. Empty bars correspond to samples for which no reads were obtained or which were removed for possessing only one DIV (considered as insufficient sequence depth). No-template controls from the DNA extraction and PCR amplification (n=3 for each) were also sequenced and are shown. Ctl - = negative control treatment where corals were chemically bleached but not reinoculated with any Symbiodiniaceae. Ctl + = positive control treatment where corals were chemically bleached and then reinoculated with freshly isolated homologous Symbiodiniaceae. RI = reinoculation treatment where corals were reinoculated with a cultured *Cladocopium* C1^acro^ strain. RiS = reinoculation with sand treatment where corals were reinoculated as in treatment Ri in the presence of sterilised sand. N = native Symbiodiniaceae community before bleaching. * = dead corals.
